# Severe Anemia From Vitamin B12 Deficiency Presenting With a Craving for Bleach Powder: An Odd Case of Pica

**DOI:** 10.1155/crps/8779524

**Published:** 2025-08-22

**Authors:** Emmanuel D. Meram, Sesilia Kammo, Shea Repins, Gregory C. Mahr

**Affiliations:** ^1^School of Medicine, Wayne State University, Detroit, Michigan, USA; ^2^Department of Psychiatry, Henry Ford Hospital, Detroit, Michigan, USA

**Keywords:** anemia, bleach craving, macrocytic anemia, pica, vitamin B12 deficiency

## Abstract

Pica, the ingestion of nonnutritive substances, represents a complex and poorly understood phenomenon. Although it is inherently a psychiatric condition, it has an intricate relationship with other psychiatric, physiological, and pathological states, suggesting a highly multifactorial etiology. Recognizing and addressing pica in acute settings is crucial, as it poses significant health risks for patients, including the potential of toxic ingestion. Our presentation highlights the case of a 36-year-old woman with a complex psychiatric history who presented to the emergency department (ED) with severe symptomatic anemia. Her anemia was found to be macrocytic and a result of autoimmune-induced vitamin B12 deficiency. Further inquiry uncovered that, prior to admission, the patient exhibited a craving for smelling bleach powder that progressed to mouthing the toxic substance for more than a month. This is the first report of a case of bleach craving in a patient with vitamin B12 deficiency absent coexisting iron-deficiency. This unique presentation underscores the importance of psychiatric consultations as a part of comprehensive clinical assessments in emergency medical settings. We also suggest that pica presentations may be nuanced and thus it is critical to understand the biopsychosocial factors driving this behavior and target interventions in the appropriate medical domains.

## 1. Introduction

Pica is defined by the DSM-V as the ingestion of nonfood or nonnutritive substances for over a month [1]. Although commonly characterized as a psychiatric manifestation of physiological or pathological states, including pregnancy and iron-deficiency anemia (IDA) [1], its etiology is still not well understood. Various theories have attempted to link pica to specific biologic mechanisms. These proposed processes span from mineral deficiencies to dopaminergic dyssynchrony, suggesting that pica may arise from a combination of factors [2, 3]. Herein, we present the first report of a case of bleach craving as a result of macrocytic anemia from vitamin B12 deficiency, notable for its lack of simultaneous iron deficiency. We suggest that nuanced presentations of pica warrant thorough psychiatric evaluation in critically anemic patients, especially since the pica can involve toxic substances like bleach.

## 2. Case Report

Our patient is a 36-year-old-female with a past medical history of morbid obesity (BMI of 85), obstructive sleep apnea, vitamin B12 deficiency, vitiligo, and anemia, who presented to the emergency department (ED) following a 1-day duration of severe shortness of breath, left lower quadrant pain, and fatigue. The patient was given 4 L of supplemental oxygen via nasal cannula during transportation to the ED. Routine EKG showed nonspecific findings. Chest X-ray revealed cardiomegaly but no acute process, and echocardiogram revealed right ventricular dilation. Initial workup showed a markedly decreased hemoglobin of 3.8 g/dL and an elevated MCV of 105.2 fL (*N* = 80–100), consistent with symptomatic macrocytic anemia. Iron, ferritin, total iron-binding capacity (TIBC), folate, and troponin levels were within normal limits. The patient's labs were also notable for thrombocytopenia (platelet count = 62 K/µL; *N* = 150–450) and vitamin B12 deficiency (58 pg/mL; *N* > 180). Rare blasts were evident on peripheral blood smear, suggestive of immature hematopoiesis in the context of vitamin B12 deficiency. The patient was transfused with 5 units of packed red blood cells and admitted to the medical intensive care unit (MICU) for further evaluation and treatment.

Notably, the patient has a complex past psychiatric history of anxiety, depression, and posttraumatic stress disorder (PTSD). She was diagnosed with PTSD following a motor vehicle accident with traumatic brain injury nearly two decades ago and was subsequently hospitalized for suicidal ideation. Additionally, she had two prior psychiatric hospitalizations for depression and bizarre behavior with a questionable history of bipolar disorder. Her psychiatric conditions have been well-controlled with citalopram 20 mg daily and an aripiprazole 300 mg monthly injection.

The patient's significant psychiatric history, coupled with emerging concerns about pica due to her symptomatic anemia, led to a consultation from the psychiatry team. Upon encounter, the patient was lying calmly in bed with conversational dyspnea. She reported that she had been tasting powdered bleach for a greater than 1-month duration. She stated that she began doing this after discovering she enjoyed the smell and texture of the powder, but she denied any precipitating factor or stressor. The patient noted that she licks her finger, dips it into the bleach powder, and then places her finger on her tongue to taste it. She swishes the substance in her mouth and then proceeds to spit it out. She subsequently rinses her mouth with water. The patient denied ever swallowing the substance but reported tasting the bleach powder 2–3 times a day. There is no specific pattern of use, and the patient denied any other history of pica-like behavior. She was neither concerned nor bothered by her behavior but indicated her family had expressed significant concern. They have tried to dissuade her from engaging in this behavior to no avail. The patient stated she has no craving for any other substance and denied any history of suicidal attempts or nonsuicidal self-injurious behavior. The remainder of the patient's psychiatric examination was unremarkable. Social history was significant for unemployment, occasional alcohol use, marijuana tea consumed three times per week, and a 17-year history of smoking (5–6 cigarettes per day).

The patient was transferred from the MICU and monitored in the general unit over the next 3 days. She showed significant improvement in symptoms over this period (Hgb repleted to 7.9), and she elected to pursue upper endoscopy for potential gastritis in an outpatient setting. Subsequent lab work revealed positive intrinsic factor antibodies, strongly suggesting a cause for her vitamin B12 deficiency. She was instructed to begin taking vitamin B12 500 mg and pantoprazole 40 mg tablets once daily and was discharged with instructions for follow-up. The patient was subsequently lost to follow-up, thus limiting assessment of symptom resolution and change in laboratory values.

## 3. Discussion

Pica has long been associated with micronutrient deficiencies. Historically, ingestion of nonnutritive substances has been viewed either as a compensatory response to nutrient deficiency or as a factor that disrupts mucosal absorption, thereby causing the deficiency [2]. In our case, anemia was established as a direct result of autoimmune-induced vitamin B12 deficiency. Although pica has been reported in patients with reduced vitamin B12 levels, its presence has been characterized only in patients who had concurrent IDA [4]. In the same report, those with sole IDA (i.e., without any vitamin B12 anomaly) had an even greater incidence of pica than those with both types of nutrient deficiencies [4]. Other studies have not shown a significant relationship between pica and vitamin B12 deficiency [5]. Notably, although our patient has a history of iron deficiency treated with iron supplementation, her current presentation lacked the typical identifiers of iron deficiency (i.e., low iron, low ferritin, and elevated TIBC), making this case novel in its presentation.

Nevertheless, vitamin B12 is critical in maintaining neuropsychiatric equilibrium. Vitamin B12 deficiency can lead to several cognitive and behavioral symptoms, including labile mood swings and psychotic states [6, 7]. Some studies have even suggested that psychiatric presentations may be the initial and only presenting feature of vitamin B12 deficiency [6]. As such, prompt identification of these clinical presentations is crucial in establishing baseline behavior, precipitating factors, and long-term treatment plans, especially when underlying culprits can include relatively rare autoimmune conditions (e.g., pernicious anemia) that may go unnoticed. Pernicious anemia, as seen in our presentation, necessitates lifelong vitamin B12 supplementation to reverse the anemia and alleviate corresponding psychiatric symptoms [8]. It is important to note that coinciding autoimmune conditions like vitiligo may increase pretest probability for pernicious anemia [8] and should be considered as adjunctive guidance when compiling clinical differentials.

The varied clinical profiles of patients presenting with pica suggests a need to delve into the psychosocial underpinnings of the behavior. Pica is fundamentally an eating disorder marked by obsessive and illogical cravings. Some have postulated dopamine depletion to be a significant contributing factor [9]. As such, antipsychotic medications, which our patient was on for maintenance therapy, may significantly contribute to her pica manifestations by promoting dopamine loss and thereby decreasing threshold for the behavior. In addition, long-standing depression, also seen in our case, may play a crucial role in influencing pica behavior during periods of increased stress [10, 11]. Patients, however, may not always identify specific stressors that contribute to current behaviors; therefore, thorough history-taking is crucial when evaluating complex biopsychosocial presentations. Notably, the best defined comorbid psychiatric condition with pica has been obsessive–compulsive disorder, and some suggest that pica may lie within the broader spectrum of the disorder [12].

Pica has been consistently associated with various micronutrient deficiencies, including lower levels of hemoglobin and zinc. These associated deficiencies have been found among individuals exhibiting geophagy (earth consumption), pagophagy (ice consumption), and amylophagy (raw starch consumption) [2], suggesting that micronutrient imbalance may be a foundational driver across pica's varied forms. Zinc deficiency, in particular, highlights the importance of assessing vitamin and mineral status in individuals with pica. In one study, 54% of subjects suffering from pica were found to have zinc deficiency, and there was significant symptom improvement following zinc supplementation [13]. Deficiencies in calcium and magnesium have also been observed in some cases, potentially influencing neuromuscular function and cravings linked to pica [14, 15]. Given these varied associations, comprehensive assessment of vitamin and mineral status is critical to guide targeted interventions. Clinical evaluation should be tailored to the specific pica behavior; for instance, lead levels should be assessed in cases of paint or chalk ingestion, while basic metabolic panels may be necessary for geophagy-related clay consumption [1, 16]. As research continues, integrating findings on multiple nutrient deficiencies will be essential to centralize a mechanism for pica's heterogeneous causes and manifestations.

Identifying pica in patients presenting with more acute medical complications poses a challenge for clinicians. For example, life-threatening hemoglobin levels (i.e., levels below 6.5 g/dL) necessitate appropriate clinical workup and treatment, including blood transfusion. Comorbid presentations can often include chest pain or shortness of breath, and it is essential to rule out acute coronary syndrome in the emergency setting before further psychiatric evaluation. Yet, screening for pica in at-risk individuals, or even in cases of mild suspicion, may help identify cases of potentially toxic ingestion that can go unaddressed, allowing a means for proper counseling. Patients with more chronic hematologic conditions, like sickle cell disease, have greater rates of underreporting pica [17] and may warrant focused assessment to help identify health-threatening behaviors. We also suggest that physicians inquire into associated and more subtle indicators of pica, such as olfactory cravings. Desiderosomia is a recently described phenomenon of an excessive desire to smell certain odors, and it is commonly found in patients presenting with pica from IDA [18]. Our patient endorsed some aspects of olfactory craving, explicitly stating that her enjoyment of the smell and texture of the bleach powder precipitated the tasting of it. Timely detection of pica through direct inquiries is therefore crucial to preventing additional complications in at-risk and vulnerable populations.

Presentations of pica in the literature have been diverse and complicated. These broad descriptions have led to difficulty uncovering the exact cause of pica. Our presentation of pica is the first of its kind, notable for the underlying macrocytic anemia from vitamin B12 deficiency, as well as the highly toxic nature of the ingested material. Psychiatric comorbidities added to the complexity of the case, and psychiatric consultation proved valuable in the overall assessment.

## Data Availability

The data that support the findings of this study are available from the corresponding author upon reasonable request.
